# Current Knowledge and Future Challenges in Takotsubo Syndrome: Part 1—Pathophysiology and Diagnosis

**DOI:** 10.3390/jcm10030479

**Published:** 2021-01-28

**Authors:** Elias Rawish, Thomas Stiermaier, Francesco Santoro, Natale D. Brunetti, Ingo Eitel

**Affiliations:** 1 Medical Clinic II (Cardiology/Angiology/Intensive Care Medicine) University Heart Center, 23538 Lübeck, Germany; elias.rawish@uksh.de (E.R.); thomas.stiermaier@uksh.de (T.S.); 2 DZHK (German Centre for Cardiovascular Research), 23538 Lübeck, Germany; 3 Department of Medical & Surgery Sciences, University of Foggia, 71121 Foggia, Italy

**Keywords:** takotsubo syndrome, broken heart syndrome, acute heart failure, biomarker, inflammation, lipotoxicity

## Abstract

First recognized in 1990, takotsubo syndrome (TTS) constitutes an acute cardiac condition that mimics acute myocardial infarction commonly in the absence of obstructive coronary artery disease; it is characterized by temporary left ventricular dysfunction, regularly in a circumferential apical, midventricular, or basal distribution. Considering its acute clinical presentation, coronary angiography with left ventriculography constitutes the gold standard diagnostic tool to exclude or confirm TTS. Frequently, TTS is related to severe emotional or physical stress and a subsequent increased adrenergic stimulation affecting cardiac function. Beyond clinical presentation, epidemiology, and novel diagnostic biomarkers, this review draws attention to potential pathophysiological mechanisms for the observed reversible myocardial dysfunction such as sympathetic overdrive-mediated multi-vessel epicardial spasms, microvascular dysfunction, the direct toxicity of catecholamines, lipotoxicity, and inflammation. Considering the long-term prognosis, further experimental and clinical research is indispensable to elucidate further pathophysiological mechanisms underlying TTS before randomized control trials with evidence-based therapeutic management can be performed.

## 1. Introduction

**Figure 1:**
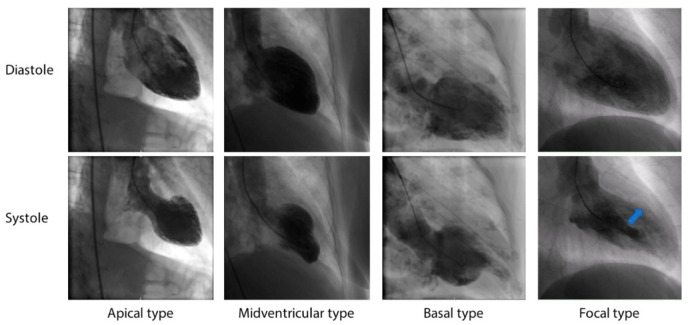
Cardiac ventriculography of different takotsubo syndrome types during diastole and systole. Hypokinetic region in focal type labeled with an arrow.

First recognized by Sato et al. in 1990 [1], takotsubo syndrome (TTS) constitutes an acute cardiac disease entity with severe left ventricular (LV) dysfunction that characteristically improves spontaneously within days or weeks. The clinical presentation mimics acute myocardial infarction (AMI), including clinical symptoms, electrocardiogram changes, and cardiac biomarkers, but it typically occurs in the absence of significant obstructive coronary artery disease, which explains the extent of LV dysfunction. Importantly, the main feature of TTS is the regional LV wall motion abnormality (RWMA), which has a characteristic circumferential pattern that results in a salient ballooning of the LV during the systole [2]. The RWMA ranges beyond the coronary artery supply regions and is predominantly localized to the apical segments of the LV, leading to the form of systolic left ventriculogram similar to the shape of a takotsubo, a Japanese octopus trap [3]. However, midventricular, basal, and focal left ventricular contractile abnormalities have been described as well [3,4] (Figure 1). The incidence of non-apical variants varies from 8% to 40%, with the prevalence of the midventricular type being at least 20%, and the basal type being about 3% [5]. Moreover, TTS can present with biventricular [6] or isolated right ventricular (RV) dysfunction [7], and it may even appear as a global LV dysfunction in up to 1.5% of patients [8]. RV involvement is present in approximately 33% of TTS patients and constitutes a predictor for poorer prognosis [9]. However, the true frequency of the isolated RV form is elusive as isolated RV dysfunction is often overlooked in clinical echo routines [10]. Indeed, echocardiography was shown to detect only 52% of patients who displayed RV-WMA with cardiac magnetic resonance (CMR) [11].

Numerous trigger factors have been found to lead up to the onset of TTS in about 70% of TTS patients [12]. Emotional stressors such as the unexpected death of a close relative may trigger the TTS, which has led to the alternative term ‘broken heart syndrome’ [13]. However, emotional triggers do not need to be negative because positive emotional experiences can trigger TTS as well (e.g., becoming grandmother, surprise farewell celebration, and positive job interview); thus, this entity can also be called the ‘happy heart syndrome’ [14]. Furthermore, manifold physical triggers, from severe medical conditions such as end-stage chronic obstructive lung disease [15], intracranial hemorrhages [16], and coronavirus disease-2019 (COVID-19) [17], to physiological processes, such as sexual intercourse [18] and pregnancy [19], may trigger TTS. Remarkably, acute coronary artery obstruction may trigger TTS as well [20]. Overall, physical triggers are more frequent than emotional stress factors (36.0% vs. 27.7%, respectively) [4]. Unfortunately, TTS is still considered to be underdiagnosed, with an underestimated risk and, despite extensive research, imperfectly understood pathogenesis [21]. Thus, the aim of the present review was to illustrate diagnostic tools and elusive pathophysiological mechanisms, delineating future directions and promising experimental approaches that may further illuminate the pathophysiology of TTS.

## 2. Clinical Manifestation and Outcome

The most frequent symptoms of TTS are chest pain (76%), dyspnea (47%), and syncope (8%) [4]. Cardiac arrest, cardiogenic shock, and severe arrhythmias occur more infrequently in patients with TTS, but they are potentially life-threatening complications [4]. However, asymptomatic TTS may be incidentally diagnosed by new electrocardiographic alterations or a rise of cardiac biomarkers [22].

The clinical presentation of TTS triggered by severe physical stress may be superimposed by the symptoms of the underlying acute disease. Indeed, patients with ischemic stroke or seizure-caused TTS have been shown to have less common chest pain [23,24] which could be referred to reduced consciousness, neurologic complications, or an abrupt hemodynamic impairment [10]. On the contrary, patients suffering emotional stress factors were shown to display a higher incidence of chest pain and palpitations [25]. In addition, patients suffering from non-apical TTS were shown to present a distinct clinical phenotype [3]. These patients were younger, had a less impaired LV ejection fraction (LVEF) and lower brain natriuretic peptide values, suffered more often from neurologic comorbidities, and had more common ST-segment depression compared to apical TTS [3,26]. The basal phenotype has been related to adrenaline-induced TTS [27], the presence of pheochromocytoma [27], and subarachnoid hemorrhage [28].

With respect to the prognosis, TTS was originally thought to be a nonthreatening disease [29]. However, more recent studies have yielded higher mortality rates in TTS patients than expected before, as long-term mortality surpassed that of patients with ST-segment elevation myocardial infarction (STEMI) [26]. In particular, a high Killip class upon admission, male sex, and diabetes mellitus were recognized as independent predictors of mortality in TTS patients [26]. Furthermore, the N-terminal pro-B-type natriuretic peptide (NT-proBNP) level at admission constitutes a predictor for short- and long-term complication TTS patients and, therefore, may be a helpful indicator for initial risk assessment [30]. An analysis of 1750 TTS cases showed a 30-day mortality of 5.9% and a long-term mortality rate of 5.6% per patient per year [4]. Major adverse cardiac and cerebrovascular events, as well as in-hospital deaths, were found to be more frequent in men than in women with TTS [4]. A long-term follow-up study yielded an increased rate of mortality and severe cardiac and cerebrovascular events beyond 30 days in men [31]. Additionally, diabetes was recognized to be correlated with heightened longer-term mortality and as an independent predictor of unfavorable outcome irrespective of further risk factors [32]. Regarding different types of TTS, in-hospital complication rates, and long-term mortality were reported to be comparable between typical and atypical types [3]. Ghadri et al. noticed an LVEF < 45%, neurologic disorders, and atrial fibrillation as independent predictors of death after adjustment for confounders. Physical triggers have also been identified to worsen the long-term prognosis [33].

Notably, a multicenter study over 1000 patients from the German and Italian Stress Cardiomyopathy (GEIST) registry yielded four variables as independent predictors of in-hospital complications: a history of neurologic disorders, RV involvement, LVEF, and male sex [34]. Therefore, the GEIST prognostic score may be helpful in early risk stratification. In addition, a recently published analysis of the GEIST registry has revealed dyspnea at admission as an independent risk factor for in-hospital complications and poor long-term outcomes [35]. Thus, symptom evaluation including dyspnea may constitute a useful tool to enhance risk-stratification models for TTS patients [35].

In summary, a distinction between AMI and TTS based on clinical presentation is not feasible. The prognosis of TTS ranges from a rapid recovery to poor early and long-term outcomes. Thus, risk stratification considering physical stressors, comorbidities (i.e., diabetes), a high Killip class upon admission, right ventricular involvement, and LVEF is essential in order to identify patients at risk.

## 3. Epidemiology

Approximately 1–2% of all patients and up to 10% of women attending clinics with acute coronary syndrome (ACS) have been diagnosed with TTS [5,36,37]. With respect to the prevalence of TTS among all hospitalized patients, Deshmukh et al. showed a rate of 0.02% [38]. Nevertheless, the prevalence is still considered to be underestimated due to the unawareness of the disease [39]. However, with the growing awareness and availability of invasive coronary angiography (CAG), a 20-fold increase in the incidence of TTS from 2006 to 2012 has been detected in the United States [40]. 

Around 90% of TTS patients are women, with a mean age of 68.5 years, and about 80% are older than 50 years [4,41]. Indeed, women older than 55 years have 4.8 times higher odds for suffering TTS than younger women. Furthermore, women have a 10.8-fold higher odds for TTS than men [38]. Interestingly, emotional triggers occur more frequent among women, while physical triggers occur more often among men [4]. Elderly patients were reported to display a higher prevalence of poorer in-hospital cardiovascular outcomes due to a higher prevalence of acute heart failure, severe arrhythmias, cardiogenic shock, and stroke [42]. TTS has also been described in children and newborns, and regarding the female predominance in adult population, a recent small study revealed an equally distribution between boys and girls in the pediatric age group [43]. Furthermore, pediatric patients more often develop heart failure symptoms or a loss of consciousness. Additionally, a high amount of younger patients display non-apical TTS variants and more severe LV impairments [44]. However, much still remains to be addressed regarding TTS in children, as there are no data concerning long-term outcomes in pediatric patients with TTS [44]. 

Even though there has been a lack of large-scale studies concerning the impact of ethnicity in TTS presentation and outcomes, a study with a total of 206 TTS patients displayed ethnical disparities; African-Americans who suffered TTS presented less frequently with chest pain or depression and anxiety as comorbidities [45]. Moreover, physical stressors appeared to be more common than in Hispanics and Caucasians [45]. Asians and African-Americans may additionally have a higher risk for complications, e.g., acute respiratory failure, requiring mechanical ventilation, or stroke compared to Hispanics and Caucasians [45]. With respect to electrocardiographic differences, African-Americans present more commonly with T-wave inversions and a more prolonged QTc, while non-African-Americans patients display more frequent ST depressions [46]. 

The reported recurrence rate of TTS ranges from 0% to 22%, depending on the duration of follow-up [5]. A systematic review by Singh et al. showed an annual recurrence rate of 1.5% [47]. In patients younger than 50 years, the recurrence rate has been found to be higher than in those older than 50 years, as triggering events are more likely to recur [48,49].

## 4. Diagnosis

Differentiation between TTS and AMI is often challenging due to their similarity in clinical presentation, electrocardiography (ECG) abnormalities, and cardiac biomarkers [50]. Therefore, CAG is considered to be the gold standard diagnostic tool to assess coronary status to rule out AMI. Commonly, echocardiography constitutes the first-line imaging tool in patients with suspected TTS for the assessment of RWMA. However, RWMA assessment can also be achieved by ventriculography [10]. There have been several criteria postulated to differentiate TTS, and the Heart Failure Association diagnostic criteria for takotsubo syndrome are the well-established ones (Table 1) [51]. Table 2 summarizes typical findings using the following diagnostic tools in TTS.

### 4.1. ECG Patterns

Common ECG findings in TTS are ST-segment elevation (44%) and T inversion (41%), mostly in the precordial leads [52,53]. When comparing STEMI and STE-TTS, Frangieh et al. demonstrated that ST-elevation (STE) in –aVR was distinctive of STE-TTS with a positive predictive value (PPV) of 91% and a negative predictive value (NPV) of 62% [54]. Meanwhile, STEMI was characterized by STE in aVR (PPV of 85% and NPV of 59%) and ST-depression in V2–V4 (PPV of 100% and NPV of 76%) [54]. However, there are a lack of prospective data, and, therefore, access to STEMI-guideline-based coronary angiography should not be delayed because of prolonged ECG analysis [5]. Other electrocardiogram findings in TTS patients include QT interval prolongation, implicating the risk of torsades de pointes tachycardia [55] and T-wave inversion, which is more frequent than in AMI and may last for months even after the recovery of LV-function [56]. Meanwhile, pathologic Q-waves and ST-depression are less often detectable in TTS than STEMI [4,57].

Dynamic changes in the ECGs of TTS patients have prognostic value. It is crucial to perform serial ECGs from admission to 72 h after. Indeed, 60% of patients develop QT interval prolongation after 72 h. Dynamic increases of QTc intervals after hospitalization are associated with better prognoses. On the other hand, prolonged QTc intervals at admission could be rated to a higher risk of cardiovascular rehospitalization at follow-up [58]. Moreover, a persistent ST elevation after 72 h from admission can be found in 19% of patients and may predict in-hospital complications [59].

### 4.2. Biomarkers

Compared with patients with STEMI, patients with TTS typically present lower levels of creatine kinase-MB (CKMB) and lower peak levels of cardiac troponin due to a relatively mild or lack of tissue necrosis [4,60]. However, upon admission, troponin values in TTS usually do not differ from those in AMI [4,60]. Furthermore, a discrepancy between the extent of biomarker elevation and the degree of angiographic myocardial dysfunction is characterizing for TTS [12]. The troponin T/CKMB ratio has been identified as a suitable parameter to discriminate TTS from AMI [61]. As serum B-type natriuretic peptide (BNP) or NT-proBNP levels are significantly elevated in response to myocardial stretch during the acute phase of TTS, Fröhlich et al. were able to demonstrate that a higher NT-proBNP/troponin T ratio could distinguish TTS from STEMI (specificity of 95% and sensitivity of 91%) and NSTEMI (specificity of 95% and sensitivity of 83%) [62]. Further underlining the value of cardiac biomarkers in the early diagnosis of TTS, Dagrenat et al. recently developed a score enabling sufficient distinction between TTS and STEMI by assessing age, gender, history of psychiatric disorders, LVEF, and BNP/troponin I ratio at admission (sensitivity of 92% and specificity of 77%) [63]. Notably, NT-proBNP levels correlate with both the extent of catecholamine increase and the severity of LV systolic dysfunction [64], and NT-proBNP levels are higher in patients with typical TTS than in patients with atypical TTS [3].

Beyond these established biomarkers, several novel markers have been proposed for differentiating TTS from AMI [65]. For instance, copeptin (C-terminal provasopressin), that is mainly synthesized in the paraventricular neurons of the hypothalamus and in the supraoptical nucleus [66], has levels that are significantly increased in patients suffering an AMI, supporting the use as a diagnostic biomarker [67,68]. Meanwhile, copeptin itself is not able to significantly distinguish TTS from AMI [69], but the copeptin/NTproBNP ratio was recently shown to discriminate TTS and STEMI in a small study [70]. 

The soluble suppression of tumorigenicity 2 (sST2), part of the interleukin (IL)-1 receptor family, was shown to predict TTS in patients admitted to the intensive care unit (ICU) [71], and, intriguingly, it has recently been reported that sST2 and soluble thrombomodulin (sTM) plasma concentrations can distinguish between TTS and acute anterior STEMI patients [72]. sTM levels in TTS patients are increased, pointing to the pathophysiological relevance of endothelial cell damage in TTS, as discussed below [73]. 

At admission, the plasmatic levels of IL-6 are lower in TTS patients compared to AMI, patients while IL-7 levels are elevated [74]. Santoro et al. reported similar data on IL-6 and thereby showed that enhanced levels of anti-inflammatory interleukins (IL-2, IL-4, and IL-10) could also be detected during the acute phase of TTS [75].

With respect to further stress responsive cytokines, the ability of growth differentiation factor-15 (GDF-15) to distinguish TTS from STEMI was shown by Stiermaier et al. [76]. In particular, biventricular ballooning was correlated to high GDF-15 concentrations, whereby GDF-15 level at admission was identified as a predicting marker of poor clinical outcome [76]. Tarantino et al. showed that levels of chromogranin-A, a marker of sympathoadrenal activation, are lower in the acute TTS than in STEMI, pointing to a greater cardiac than adrenal catecholamine release [77].

Furthermore, Jaguszewski et al. postulated a signature of four circulating microRNAs as a strong biomarker to differentiate TTS from STEMI; the signature comprises miR-26a, miR-1, miR-133a, and miR-16, and it was found to be able to distinguish TTS from healthy and STEMI patients, with a specificity of 79% and a sensitivity of 74% for TTS vs. healthy subjects, as well as a specificity of 70% and a sensitivity of 97% for TTS vs. STEMI patients [78]. Interestingly, elevated levels of miR-16 and miR-26a have been associated with stress and affective disorders before [79,80,81]. Accordingly, a high prevalence of psychiatric disorders has been described in TTS patients [4].

In addition, a more recent microarray analysis identified A2M, ALB, APOB, APOE, C3, MFGE8, and SAA1 as hub genes of TTS that might be future diagnostic biomarkers or molecular targets for the treatment of TTS [82]. However, the authors concluded that these key genes and linked signaling pathways need further experimental verification due to the defects of analytical methods and sample sizes [82]. 

Taken together, numerous biomarkers have been proposed for the differential diagnosis of TTS, but none of these could render invasive coronary angiography redundant. Nevertheless, a recent microarray and circulating microRNA analysis have revealed promising targets for further research to support physicians in the diagnosis of TTS.

### 4.3. Angiography and Ventriculography

Even though echocardiography constitutes the first-line imaging tool in patients with suspected TTS, CAG is of decisive diagnostic importance to rule out alternative diagnoses [83]. As just recently reported by Napp et al., 23% of 1016 TTS patients had a coexisting obstructive coronary artery disease (CAD), and 41% had non-obstructive CAD [84]; thus, a meticulous comparison of biplane ventriculography and CAG is required in order to identify a perfusion–contraction mismatch [85,86] that distinguishes TTS from classical AMI [81]. Notably, Desmet et al. demonstrated that about 33% of patients with typical apical ballooning display a very small area with preserved contractility in the most apical part of the left ventricle, which is called ‘apical nipple sign’ [87] and constitutes a useful tool to distinguish TTS from anterior STEMI, in which this sign is not detectable. Furthermore, spontaneous coronary artery dissection (SCAD) should be included in the differential diagnosis of patients suspected of having TTS and coronary angiograms inspected for subtle SCAD [88,89]. TTS and SCAD may coexist. Several possible causal links have been discussed, as TTS in SCAD could be triggered by ischemia and subsequent physical stress caused by SCAD [89]. On the other hand, it has been hypothesized that excessive contraction of the LV base accompanied by apical ballooning in TTS could serve as an anatomic or functional substrate for the causation of SCAD [90]. Nevertheless, this hypothesis needs to be verified in future studies. Intravascular imaging tools, such as intravascular ultrasound or optical coherence tomography (OCT), are useful to detect other types of MINOCA (myocardial infarction and no obstructive coronary artery disease) such as plaque rupture or erosion [91]. Additionally, as LV outflow tract obstruction (LVOTO) occurs in about 20% of TTS patients [92], intraventricular pressure gradients should be assessed.

### 4.4. Echocardiography

Echocardiography constitutes the first-line imaging modality to assess alterations in ventricular function such as RWMAs, RV involvement, hemodynamic status, mechanical complications (LVOTO), pericardial effusion, LV thrombi, and pulmonary artery systolic pressure [93]. In contrast to apical and midventricular TTS, the diagnosis of uncommon forms is challenging when using echocardiography alone; thus, the focal type should be suspected only after exclusion of other possible etiologies [83]. Importantly, a reduction in LVEF upon admission as well as RV involvement are related to poorer in-hospital outcomes [94,95]. Furthermore, advanced systolic dysfunction was recently suggested to worsen long-term prognosis [95]; thus, LV and RV function should be carefully defined [83].

RWMA and LVEF commonly recover within a few weeks, while the impairment of global longitudinal strain, untwist rate, and time to peak untwisting have been reported to persist for weeks to months after acute TTS [96,97], possibly contributing to the long-term prognosis of patients that suffer TTS.

Finally, contrast echocardiography may be used to improve RWMA and ventricular thrombus detection [83], while 3D echocardiography may be a useful to improve the assessment of RV involvement [98].

### 4.5. Cardiac Magnetic Resonance Imaging

**Figure 2:**
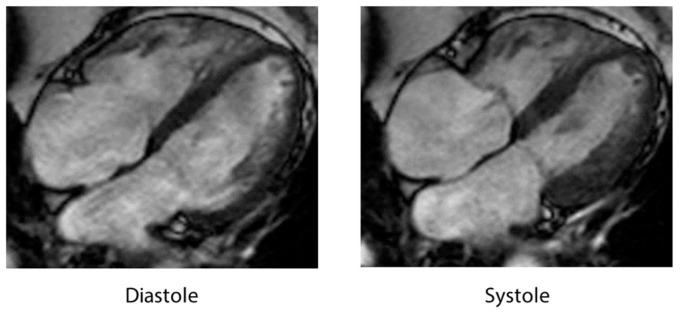
CMR images (four-chamber view) demonstrating biventricular ballooning in TTS, thus indicating RV involvement.

CMR constitutes the gold standard for the qualitative and quantitative assessment of RWMAs and the accurate quantification of LV and RV volumes and function, thus allowing for a comprehensive evaluation of structural and functional abnormalities in patients with TTS [9]. Further pathologies, including pleural effusion, pericardial effusion, and intraventricular thrombi, can also be precisely analyzed using CMR [9,99]. Ventricular morphology and function should be assessed by balanced, steady-state free precession imaging including short-axis plane and three long-axis planes (two-chamber, four-chamber, and LVOT view) [83]. Interestingly, CMR imaging was found to have a significantly decreased left atrium (LA) function during the acute/subacute phase of TTS compared to anterior STEMI [100]. However, the impairment of LA performance seemed to be transient in TTS with recovery during follow-up [100]. CMR-feature tracking (FT) is considered to be an emerging tool for the quantitative analysis of regional LV deformation corresponding to speckle tracking echocardiography, thus affording the quantification of biatrial and biventricular measures of deformation, i.e., strain, dyssynchrony, and torsion [101]. Indeed, global longitudinal strain has been reported to be able to serve as a potential determinant of outcome in TTS [102]. Furthermore, impaired rotational mechanics and transient circumferential dyssynchrony have been identified as characteristic CMR-FT features in TTS [102]. RV myocardial strain analysis using CMR-FT allows for the exact evaluation of RV involvement in TTS (Figure 2). These novel parameters may help to identify high-risk patients, though this needs to be validated in prospective studies [102].

**Figure 3:**
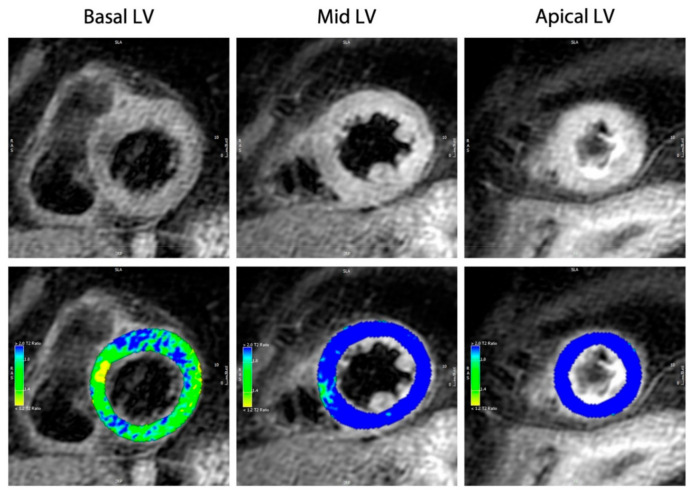
T2-weighted images (short-axis view) demonstrating normal signal intensity (SI) of the basal myocardium and global edema of the mid and apical myocardium. SI analysis (bottom row) of the T2-weighted images with color-coded display of relative SI normalized to skeletal muscle (blue indicates an SI ratio of myocardium to skeletal muscle of ≥1.9 or higher, thus indicating edema; green/yellow indicates a normal SI ratio of <1.9).

Myocardial tissue characterization constitutes a major advantage of CMR due to its ability to discriminate reversible from irreversible myocardial injury; the absence of late gadolinium-enhancement (LGE) in dysfunctional LV regions—which is defined as a diagnostic criterion for TTS [9]—indeed allows for the distinction between TTS and MI (transmural or subendocardial LGE related to a vascular territory) and most cases of acute myocarditis (commonly epicardial or patchy LGE) [2,81,103]. However, the usage of a low LGE signal intensity (SI) threshold (three standard deviations (SD)) have yielded subtle focal or patchy LGE in patients with TTS, whereby a SI threshold of five SDs above the mean did not result in areas of LGE in TTS patients [9]. An immunohistologic analysis by Rolf et al. took a disproportionate rise of extracellular matrix rich in collagen-1 due to transient fibrosis into account for the presence of LGE in TTS patients [104]. With respect to the prognostic relevance of LGE in TTS patients, a small study revealed increased disease severity and prolonged recovery of affected patients [105], whereas a multicenter prospective registry has not yielded any differences in prognosis of patients with and without LGE [9]. The detection of edema in TTS patients—conceivably due to inflammation, increased wall stress, or transient ischemia—is detectable in regions with abnormal systolic function when using T2 weighted imaging [9,106] (Figure 3). It is a temporary and reversible phenomenon that is characteristically encountered during the acute phase, thus enabling the in vivo assessment of the acuteness, severity, and extent of myocardial stunning in TTS, and it fades within few weeks along with the recovery LV-function [83,106]. 

CMR should be used in the acute phase of TTS, when echocardiographic images are suboptimal, or when differential diagnosis such as myocarditis requires a different therapeutic strategy [83]. In the post-acute phase, CMR is obligatory in all patients within two months, particularly in order to confirm the diagnosis of TTS [83].

## 5. Pathophysiology

### 5.1. Sympathetic Hyperactivity

**Figure 4:**
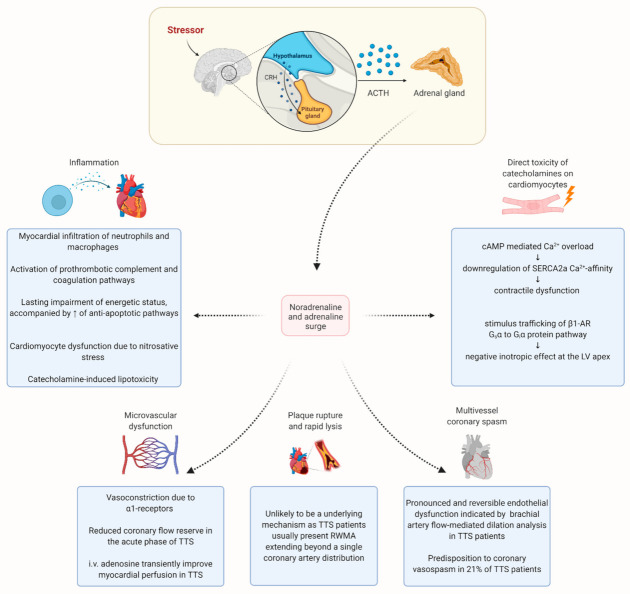
Pathophysiological mechanisms in TTS. CRH: corticotropin-releasing hormone; ACTH: adrenocorticotropic hormone; cAMP: cyclic adenosine monophosphate; SERCA2a: sarco/endoplasmic reticulum Ca^2+-^ATPase; β1-AR: β1-adrenoceptor; LV: left ventricular; RWMA: regional LV wall motion abnormality.

Though the exact underlying pathophysiological mechanisms of TTS are still elusive, the pivotal importance of sympathetic hyperactivity is generally accepted [10] (Figure 4). Increased release of (nor)epinephrine is initiated by the cognitive centers of the brain through the activation of the hypothalamic–pituitary–adrenal (HPA) axis in response to a given stress, which is named HPA gain [5]. Indeed, Wittstein et al. showed massive increased concentrations of plasma catecholamines and stress-related circulating neuropeptides in the acute phase of TTS compared to patients with STEMI [107]. However, these exceeding levels of catecholamines were not confirmed in a recent meta-analysis including 108 patients [108] and were thus attributed to a miscalculation of the catecholamines in the Wittstein study [109]. Due to the short half-life of catecholamines, it seems plausible that the plasma levels of catecholamines are very high at the inception of the human TTS but already normalized or only moderately elevated after hospitalization, as recently shown by Madias [108]. Nevertheless, elevated levels of norepinephrine have been detected in in the coronary sinus of TTS patients, pointing to an enhanced release of myocardial catecholamines in TTS [110]. Accordingly, the various ballooning patterns and clinical features of TTS can be precipitated by the administration of beta-agonists and catecholamines in patients [111]. Further underlying the role of adrenergic stimulation in TTS, Ueyama et al. demonstrated the ability of alpha- and beta-receptor blockades to attenuate LV apical ballooning triggered by immobilization in rodents [112]. In addition, the administration of different catecholamines was able to induce different RWMA patterns by an afterload-dependent mechanism in a more recent rat model [113]. Beyond the surplus of catecholamines, microneurographic studies have revealed direct evidence for sympathetic hyperactivity, as increased sympathetic nerve activity was detected in TTS patients [114]. Furthermore, myocardial 123I-metaiodobenzylguanidine (^123^I-MIBG) scintigraphy have shown a functional alteration in presynaptic sympathetic neurotransmission in patients with TTS [115]. These abnormalities may last for months after the recovery of LV function and are suggested to promote an interstitial mononuclear inflammatory response and occasionally contraction band necrosis, constituting a pathological hallmark of TTS [107,116]. Further underlining the relevance of sympathetic nervous system in pathogenicity of TTS, myocardial damage was reduced in rats whose spinal cords were severed at C7–T1 before adrenaline administration, indicating that the administered catecholamine did not lead to myocardial lesions, but adrenaline could instead act as trigger for local cardiac sympathetic disruption [117,118]. In accordance, the circumferential pattern of RWMA does not usually match with the coronary artery supply region but frequently correlates with the pattern of the myocardial sympathetic nerve terminals [119].

Overall, increased sympathetic stimulation is crucial to TTS. However, the exact mechanism by which a catecholamine surge orchestrates myocardial stunning in the diversity of regional ballooning remains elusive; thus, several pathophysiological theories are discussed below.

#### 5.1.1. Multivessel Coronary Spasm

Epicardial spasm due to sympathetic hyperactivity has been proposed as a possible underlying mechanism in TTS [10]. Indeed, mental stress has been shown to induce endothelial dysfunction, which has been prevented by endothelin-A receptor antagonism [120].

Furthermore, TTS has been associated with Raynaud syndrome and migraines, supporting the pathophysiological relevance of vasomotor dysfunction [121]. In accordance, an investigation of the brachial artery flow-mediated dilation in TTS patients revealed a pronounced and reversible endothelial dysfunction in patients with TTS, which gradually improved over several weeks [122,123]. Drawing attention to a prognostic relevance, a more recent study described a direct correlation among brachial artery flow-mediated dilation and the length of hospital stay [124]. Moreover, using intracoronary acetylcholine, Tsuchihashi et al. discovered a predisposition to coronary vasospasm in 21% of TTS patients; thus, most TTS patients do not show any evidence of epicardial spasm, even after applying provocative agents [125]. 

Additionally, myocardial bridging was proposed as a potential factor in the pathogenesis of TTS [126]. However, studies with higher number of cases have displayed no difference in the prevalence of myocardial bridging in patients with TTS compared with a matched control group [127,128]. Nevertheless, coronary artery tortuosity and a long recurrent wraparound left anterior descending artery (LAD), as well as LAD recurrent segments, were found to have a higher prevalence in TTS patients than in matched controls [127,128].

#### 5.1.2. Plaque Rupture

Plaque rupture, thrombosis, and subsequent transient ischemia followed by rapid lysis have been discussed as a mechanism for myocardial stunning in TTS. Indeed, a high prevalence of atherosclerotic plaques and highly vulnerable thin cap fibroatheromas were reported in TTS patients by using optical coherence tomography. However, ruptured plaques or intracoronary thrombi were not detected, speaking against a causal association between plaque rupture and TTS [91,129]. Besides, as outlined above, TTS patients usually present RWMA extending beyond a single coronary artery distribution.

#### 5.1.3. Microvascular Dysfunction

As delineated before, most patients presenting with TTS have angiographically normal coronary arteries or non-obstructive CAD. Thus, coronary microcirculation has been proposed as a possible actor that may play a decisive role in the pathogenesis of TTS. Indeed, coronary microcirculation, involving the coronary pre-arterioles and arterioles (< 500 μm diameter), regulates coronary blood flow in response to mechanical, metabolic, and neural factors, thus explaining its dysfunction curtail myocardial perfusion [130]. For instance, the vasoconstrictor effects of catecholamines are primarily exerted in the coronary microvasculature where α1-receptors predominate [131]. Further underscoring the hypothesis of acute microcirculatory dysfunction in TTS, myocardial biopsies have revealed the catecholamine-induced apoptosis of microvascular endothelial cells in TTS patients [132]. In addition, several studies have displayed a reduced coronary flow reserve (CFR) in the acute phase of TTS by using doppler transthoracic echocardiography, further pointing to a microvascular dysfunction as a causative mechanism [133,134,135]. However, a more recent analysis showed that, despite poorer LV systolic function, acute CFR was less impaired in TTS than in AMI, thus suggesting the involvement of other mechanisms in the pathogenesis of RWMA in TTS [136]. Nevertheless, by using the thrombolysis in myocardial infarction frame-count (TFC) technique, several studies have shown diffuse TFC abnormalities that suggest an increase of coronary microvascular resistance in patients with TTS [137,138,139]. Accordingly, the intravenous administration of adenosine transiently improved the wall motion score index, myocardial perfusion, and LVEF in the acute phase of TTS [140]. However, catecholamine hyperstimulation due to cold pressor testing (CPT) years after an acute episode of TTS has shown increased transient RWMAs that were accompanied by a significant reduction of coronary vasodilation reserve. According to Barletta et al., the noticed dissociation between CPT-induced RWMAs and regional coronary blood flow reduction argues against a mechanistic role of vasospasm of epicardial coronary vessels; thus, a persisting susceptibility to microvascular dysfunction in TTS patients is suggested [141]. Accordingly, coronary vasomotion to acetylcholine has been demonstrated to be impaired in women that had suffered TTS before [142], while an enhanced vascular reactivity and a decreased endothelial function in response to acute mental stress have been revealed in post-TTS patients [143]. In conclusion, an increased microvascular reactivity, possibly sympathetically mediated, should be considered as key player in the pathogenesis of TTS.

#### 5.1.4. Direct Toxicity of Catecholamines on Cardiomyocytes

Typical structural changes due to catecholamine overload include contraction band necrosis, an increase of the extracellular matrix, and mild neutrophil infiltration [144]. On a molecular level, Nef et al. demonstrated an enhanced ventricular expression of sarcolipin and the dephosphorylation of phospholamban in TTS patients, potentially resulting in a downregulation of sarcoplasmic-Ca^2+^-adenosine-triphosphatase (SERCA2a) gene expression and myocardial contractility dysfunction due to a decreased Ca^2+^-affinity of SERCA2a [145]. Accordingly, intensive G-protein stimulated β_1_-adrenergic receptor signaling has been identified to downregulate SERCA2a gene expression via the cyclic adenosine monophosphate responsive element binding protein-1 and nuclear factor of activated T-cells pathways [146,147].

Grippingly, in mammals, LV β-adrenergic receptor (β-AR) density is highest and sympathetic innervation is the lowest in the apex [148,149], indicating an increased vulnerability to high levels of circulating catecholamines. Indeed, excessive signaling by β1-AR and cyclic adenosine monophosphate (cAMP)-dependent protein kinase catalytic subunit α (PKA C-α) could lead to the cardiotoxicity observed in patients with TTS because of cardiomyocyte and mitochondrial calcium overload [149]. According to Lyon et al., norepinephrine stimulates myocardial β1-AR; this leads to an increased contractility at the LV base [149], whereby an epinephrine surge caused by extreme stress promotes the stimulation of β2-AR, thus leading to a molecular switch from G_s_α to G_i_α protein pathway, which is called stimulus trafficking—all of which results in a negative inotropic effect at the LV apex [150]. Therefore, apoptosis observed in the apical LV because of extreme stimulation of β1-ARs could partially be diminished by the β2-AR-G_i_ protein switch, constituting a physiological balance to prevent excessive catecholamine-mediated damage [149,151]. Hence, the stunned LV apex (due to β2-AR stimulation) is consequently exposed to the hypercontractile LV base (due to β1-AR stimulation), thus causing increased end-systolic LV pressure and leading to the apical ballooning pattern [150]. While this theory had been underscored by experimental rodent models for years [148,152], Nakano et al. just recently provided the first histologic evidence of the involvement of β-ARs alteration in the pathogenesis of TTS: β-arrestin2 and G protein-coupled receptor kinase 2 (GRK2), which initiate the alteration of β-AR signaling, were more frequently detectable in the myocardium in acute-phase TTS than in controls [153]. Considering the role of cAMP-dependent PKA activation in cardiotoxicity during TTS, milrinone—a phosphodiesterase 3 inhibitor that causes an increased activation of PKA—has been shown to induce TTS-like dysfunction in the absence of exogenous catecholamines in rodents, while pretreatment with propranolol and metoprolol diminished TTS-like akinesia in a dose-dependent manner [154].

#### 5.1.5. Genetic Predisposition

Supporting the relevance of the desensitization and downregulation of G protein-coupled receptor signaling in TTS, a L41Q polymorphism of GRK5, one of the two isoforms predominant in the heart, has been shown to render patients susceptible to TTS [155]. However, there is an ongoing debate considering the GRK5 polymorphism in TTS, as a larger Australian study failed to indicate an association [156] but a smaller Italian study showed a significant difference in the frequency of GRK5 polymorphism between TTS patients and a control group [157]. The first genome-wide association study in larger cohort of TTS showed promising preliminary results, as 18 loci containing top single nucleotide polymorphisms (SNPs) that were supported by SNPs in a high linkage disequilibrium were detected. Two out of the 18 loci contained SNPs with hits in the genome-wide association study (GWAS) catalog (traits: thyroid stimulating hormone, blood pressure) [158]. Several studies have shown an association between TTS phenotype and ß1-AR gene polymorphisms [159,160,161]. However, a multigenerational Mendelian inheritance pattern for TTS has not been described [10]. 

### 5.2. Hormonal Factors

Regarding the aforementioned high prevalence of TTS in postmenopausal females, one could suggest a hormonal influence. Indeed, a reduction of estrogen levels following menopause has been revealed to enhance the vulnerability to TTS in women [162]. Estrogens have been shown to attenuate the sympathetic response to mental stress in perimenopausal women, reduce catecholamine-induced vasoconstriction, [163,164], and regulate endothelial nitric oxide (NO) synthase in order to influence vasomotor tone [165]. Nevertheless, systematic data indicating a significant correlation between estrogen levels and development of TTS are lacking [10].

### 5.3. The Role of Inflammation in TTS

Inflammation in TTS has received growing interest in the recent years; however, the first indication of its relevance was published in a case report by Sato in 2005 that considered a 70-year-old woman suffering TTS associated with microscopic polyangiitis. In this case, anti-inflammatory therapy using (methyl)prednisolone completely resolved ventricular dysfunction [166]. Indeed, in a recent multi-center, prospective study, Scally et al. demonstrated that TTS is characterized by the myocardial infiltration of macrophages, alterations of distribution of monocyte subsets, and an increase of plasmatic pro-inflammatory cytokines. In particular, the authors combined CMR with the infusion of superparamagnetic particles of iron oxide (USPIO). These are mainly phagocytosed by activated macrophages, allowing for the non-invasive detection of monocytes. Stunningly, myocardial uptake of USPIO was higher in acute TTS than in controls, pointing to a macrophage-driven cellular infiltration in the myocardium of TTS patients. Furthermore, serum levels of pro-inflammatory cytokines CXCL1, IL-6 and IL-8 have been observed. The authors described an increase in pro-inflammatory CD14^++^CD16^–^ macrophages, as well as a decrease in intermediate CD14^++^CD16^+^ and nonclassical CD14^+^CD16^++^ monocytes. While USPIO enhancement was no longer detectable after five months, increased IL-6 levels and a decreased number of intermediate monocytes persisted, pointing to a chronic low-grade inflammation [167]. Accordingly, immunohistochemical staining revealed the infiltration of macrophages persisting up to three months after acute TTS in patients [144]. Moreover, increased admission levels of inflammatory (IL-6) and anti-inflammatory interleukins (IL-10) were related with higher risk of adverse events during follow-up [168].

To further determine the inflammatory characteristics of TTS, Wilson et al. used a well-established experimental model of stress-induced TTS initiated by isoprenaline injections in female Sprague Dawley rats, showing early neutrophil infiltration followed by macrophages after isoprenaline injection [169]. Importantly, just the proinflammatory M1 macrophage phenotype increased, but the percentage of anti-inflammatory M2 macrophages did not increase significantly; however, individual levels correlated with recovery in LV function [169]. 

Drawing further attention to the innate immune system, the expression of apoptosis morphological patterns and Toll-like receptors (TLR) were found to differ in the course of TTS-induced rodents in comparison with the controls [170]. Fitzgibbons et al. demonstrated that complement, coagulation, and inflammation pathways were intensified in TTS patients compared with controls [171]. Against the background of the well-established intimate interplay between players of the innate immune system such as complement system or macrophages and the plasmatic coagulation factors [172], these observations may serve as possible explanations for reported thromboembolic complications in the short term and aforementioned increased mortality risk in the long term after TTS [51].

In TTS patients, ^31^P-magnetic resonance spectroscopy has been used to determine the phosphocreatine/γ-adenosine triphosphate (PCr/γATP) ratio, which constitutes a well-established tool for the in-vivo measurement of myocardial energetic status [173], and data analyses have revealed a severe impairment of the energetic status at both the acute phase of TTS and after a follow-up period of several months [167]. Indeed, biopsies have shown an enhanced expression of survival pathways in cardiomyocytes, pointing to an adaptability to reduced energy production [174]. While phosphoinositide 3-kinase (PI3K) was upregulated and its counterpart phosphatase and tensin homolog (PTEN) was downregulated, Nef et al. further noticed an activation of anti-apoptotic pathways (Bax/Bcl-2-ratio), indicating a protection mechanism against myocardial apoptosis and necrosis [175]. Moreover, the enhanced expression of poly-ADP ribose (PAR), a downstream product of poly(ADP-ribose)polymerase (PARP)-1 activation, and 3-nitrotyrosine (3-NT) have been found in myocardium of patients that have died of TTS, thus indicating a plausible mechanism of energetic deficiency due to nitrosative stress in TTS [176]. Indeed, myocardial β_2_-adrenoceptor stimulation is assumed to increase NO release [177,178]. The mentioned immunohistochemical findings were recently confirmed using isoproterenol-induced TTS animal model, as the myocardial accumulation of 3-NT and PAR were reported [178]. Remarkably, the myocardial expression of pro-inflammatory α-arrestin thioredoxin-interacting protein (TXNIP) was increased in a TTS mice model, accompanied by enhanced TXNIP expression stimuli, such as a diminished NO effect [179] or the distraction of laminar flow and associated shear stress [180] due to glycocalyx shedding, which have recently been associated with TTS [181]. Fascinatingly, the PARP-1 inhibitor 3AB significantly reduced the severity of LV function impairment in a rodent model, illustrating the pivotal importance of nitrosative stress-induced pathways in the pathophysiology of TTS [178]. Accordingly, a protective effect of hydrogen sulfide in catecholamine-treated rodents, due to the alleviation of isopropanol-induced reactive oxygen species (ROS) generation, was reported [182]. Moreover, the flavonoid icariin has lately been demonstrated to prevent isoproterenol-induced TTS-like cardiac dysfunction in rats through a reduction of ROS levels, thereby suppressing TLR4/nuclear factor kappa-light-chain-enhancer of activated B cells (NF-κB) pathway expression [183].

Shao et al. revealed intramyocardial lipid droplet accumulation in rodent cardiomyocytes in response to catecholamine surge [184]. This had been confirmed in myocardial biopsies of patients suffering acute TTS but not after recovery [144]. Lipid droplets are tiny cellular organelles that control the storage and metabolism of neutral lipids. In non-adipocytes, lipid droplets protect against an excess of fatty acids (FAs) because they store surplus FAs in form of neutral triacylglycerol, as extensive lipid droplet accumulation can lead to an inflammatory response and so called lipotoxicity [185]. Indeed, Shao et al. were able to associate lipotoxicity with isoproterenol-induced LV dysfunction, including metabolic, electrophysiological, and neurogenic stunning. As the gene expression of the ApoB lipoprotein was depressed after isoprenaline injection, the authors further concluded that catecholamine surge in TTS may inhibit myocardial ApoB lipoprotein, thus leading to an impaired lipid export and cardiac lipotoxicity in TTS [184]. Using iodine-123-*β*-methyl-p-iodophenyl penta-decanoic acid (^123^I-BMIPP) and serial tallium-201 (^201^Tl) dual-isotope myocardial single-photon emission computed tomography (SPECT), Kurisu et al. indeed demonstrated that perfusion defects had a reduced extent when measured by ^201^Tl compared with ^123^I-BMIPP (evaluation of myocardial fatty acid metabolism), thus suggesting a more severe impairment of myocardial fatty acid metabolism than myocardial perfusion [138] and possibly explaining lipid accumulation in TTS due to dysfunctional fatty acid metabolism. A recently established in vitro pluripotent stem cell (iPSC) model of TTS yielded an increased expression of cAMP and the cAMP-dependent, PKA-mediated hyperphosphorylation of ryanodine receptor 2, phospholamban, troponin I, and Ca_v_1.2, leading to a reduced calcium time to transient under catecholamine-induced stress, mimicking some pathophysiological mechanisms that have been observed in acute TTS and enabling the further in vitro exploration of underlying mechanisms. In addition, TTS-iPSC-cardiomyocytes (CMs) displayed a diminished contraction force and a higher susceptibility to catecholamine-stimulated inotropy compared with controls. Intriguingly, catecholamine-treated TTS-iPSC-CMs displayed increased lipid accumulation, which was confirmed by differentially expressed the lipid transporters carnitine palmitoyltransferase-1c and fatty acid translocase [186], further underlying the role of cardiac lipid metabolism in TTS.

In summary, inflammation can be considered to be a fundamental part of the pathophysiology of TTS. However, the cause–effect relationship remains unknown. Hence, future experimental studies should focus these causative mechanisms of the disease. However, from a translational perspective, anti-inflammatory therapies constitute a promising therapeutic approach in TTS; thus, intervention studies should be considered.

## 6. Conclusions

TTS constitutes a complex acute cardiac condition mimicking AMI that usually occurs in the absence of obstructive-CAD, leading to RWMA with characteristic circumferential patterns and an impairment of LV contractility. Due to its acute clinical presentation, coronary angiography with left ventriculography is considered to be gold standard diagnostic modality to confirm or exclude TTS. However, CMR is of importance for the differential diagnosis in patients with suspected TTS when echocardiographic images are suboptimal or when the diagnosis of another type of MINOCA (e.g., myocarditis) requires a different therapeutic approach. With respect to the prognosis, the long-term mortality of TTS exceeds that of patients with STEMI. TTS is frequently associated with severe emotional or physical stress and a subsequent increased adrenergic stimulation that affects cardiac (micro)perfusion and function. Several pathophysiological mechanisms that do not reciprocally exclude each other, such as sympathetic overdrive-mediated multi-vessel epicardial spasms, microvascular dysfunction, the direct toxicity of catecholamines, and inflammation, have been proposed. The predominance of postmenopausal female among TTS patients indicates estrogen deficiency as an important predisposing factor. However, further experimental and clinical research is required to illuminate the pathophysiological mechanisms underlying TTS before randomized control trials with evidence-based therapeutic management can be done.

## Figures and Tables

**Table 1:** Heart Failure Association diagnostic criteria for takotsubo syndrome (TTS).

Diagnostic criteria	
1	Transient regional wall motion abnormalities of left ventricle (LV) or right ventricle (RV) myocardium which are frequently, but not always, preceded by a stressful trigger (emotional or physical).
2	The regional wall motion abnormalities usually ^a^ extend beyond a single epicardial vascular distribution and often result in circumferential dysfunction of the ventricular segments involved.
3	The absence of culprit atherosclerotic coronary artery disease including acute plaque rupture, thrombus formation, and coronary dissection or other pathological conditions to explain the observed pattern of temporary LV dysfunction (e.g., hypertrophic cardiomyopathy and viral myocarditis).
4	New and reversible electrocardiography (ECG) abnormalities (ST-segment elevation, ST depression, left bundle branch block (LBBB) ^b^, T-wave inversion, and/or QTc prolongation) during the acute phase (3 months).
5	Significantly elevated level of serum natriuretic peptide (B-type natriuretic peptide (BNP) or N-terminal pro-B-type natriuretic peptide (NT-proBNP)) during the acute phase.
6	Positive but relatively small elevation in cardiac troponin measured with a conventional assay (i.e., disparity between the troponin level and the amount of dysfunctional myocardium present). ^c^
7	Recovery of ventricular systolic function on cardiac imaging at follow-up (3–6 months) ^d^.

^a^ Acute, reversible dysfunction of a single coronary territory has been reported. ^b^ Left bundle branch block may be permanent after takotsubo syndrome but should also alert clinicians to exclude other cardiomyopathies. T-wave changes and QTc prolongation may take many weeks to months to normalize after recovery of LV function. ^c^ Troponin-negative cases have been reported but are atypical. ^d^ Small apical infarcts have been reported. Bystander subendocardial infarcts, involving a small proportion of the acutely dysfunctional myocardium, have been reported. These infarcts are insufficient to explain the observed acute regional wall motion abnormality.

**Table 2:** Typical findings using established diagnostic tools in TTS.

Diagnostic Tool	Finding
ECG	ST-segment elevation (particularly in -aVR), ST depression, LBBB, T-wave inversion, QTc prolongation
Biomarkers	Elevated troponin T with higher NT-proBNP/troponin T ratio than in ST-segment elevation myocardial infarction (STEMI)
Angiography and ventriculography	Absence of culprit atherosclerotic coronary artery disease including acute plaque rupture, thrombus formation, and coronary dissection, as well as characteristic regional LV wall motion abnormality (RWMA). Apical nipple sign.
Echocardiography	RWMAs
CMR (cardiac magnetic resonance)	RWMAs, RV involvement, late gadolinium-enhancement signal intensity threshold < 5 SD, and edema using T2 weighted imaging in dysfunctional LV regions.
